# A study to determine the three-dimensional (3D) facial shape characteristics for a successful FFP3 mask fit

**DOI:** 10.1038/s41598-024-80001-4

**Published:** 2024-11-19

**Authors:** Manpreet K. Gakhal, Anant Bakshi, Min Gu, Balvinder S. Khambay

**Affiliations:** 1 Department of Orthodontics, The School of Dentistry, Institute of Clinical Sciences, College of Medical and Dental Sciences, University of Birmingham, 5 Mill Pool Way, Birmingham, B5 7EG UK; 2 Discipline of Orthodontics, Faculty of Dentistry, The University of Hong Kong, Pok Fu Lam, Hong Kong SAR China

**Keywords:** Dentistry, Health services, Medical imaging

## Abstract

A reported 20% of dental staff will fail their fit test for a disposable FFP3 respirator. This needs to be factored into future pandemic workforce and PPE supply planning. At present there are no scientifically or universally accepted facial shape criteria to design and produce facial masks that will fit the entire work force. This study presents differences in facial shape, volume and surface area between individuals who passed on several FFP3 masks (pass group) and participants who passed on only one FFP3 mask (fail group). Three dimensional images of 50 individuals, 25 in each group, were taken at rest and at maximum smile using a DI4D SNAP 6200 camera system. The images were processed, and four “average faces” were produced—pass group at rest, fail group at rest, pass group at maximum smile and fail group at maximum smile. Simple Euclidian linear and angular measurements, geodesic surface distances and volume and surface area enclosed within the mask were analysed. The results of the study show that individuals who are more likely to pass a mask fit test have longer faces, wider mouths, greater geodesic surface distances and a greater volume and surface area of soft tissue enclosed within the mask boundary. This would suggest that some manufactures masks may be too large, and they need to reduce the size of their masks or produce a category of sizes, accepting the fact that one size does not fit all.

## Introduction

Prior to the COVID-19 pandemic, the use of a “filtrating face piece” (FFP) in dentistry was not mandatory when treating or coming face to face with patients. The ease, route, and rate of transmission and varied immune response of COVID-19 meant that health care workers were at high risk of becoming infected. Several aerosol-generating procedures (AGPs) in dentistry were identified as a possible route of viral transmission. Aerosols are particles < 5 μm in size that can remain suspended in the air, travel over a distance and may cause infection^1^. The risk of viral transmission can be markedly reduced by well-fitting respiratory personal protective equipment (PPE). In many NHS Trusts, professionally fitted FFP3 masks were advocated, as they have a 99% efficiency of filtering aerosols containing particles as small as 0.3–0.6 μm in diameter^2^.

Manufactures of FFP3 masks require some basic facial dimensions to design and produce facial masks that will fit the work force. These were initially based on the facial anthropometric measurements of 25 American military adult personnel^3^. Subsequently, these values were found to be no longer representative of the general working population of the USA, and additional measurements of a wider demographic were collected^4^. In total, 18 facial dimensions were collected, based on traditional anthropometric methods, of 3997 individuals recruited from the construction, firefighting, healthcare, law enforcement and manufacturing industries from 41 sites across 8 states; the databases came to be known as “*respirator fit test panels*”.

The facial dimensions used for mask design were based on a bivariate model – two measurements, which in the case of half mask facial respirators, were face height and lip length and for full-face masks, were face height and face width. The bivariate model for mask construction was based on the mean minimum facial height and facial width for females minus two standard deviations (SD) and the maximum based on males plus two SDs. Facial length has been reported as a factor that varies between passing and failing a mask fit test^4–6^. In addition, nasal protrusion^4–8^ and facial width^4,8,9^ have also been reported as important facial dimensions. A significant correlation between mask fit and mouth width has also been reported^9^. However, some studies have reported no association between any facial measurements and respirator fit^10^. This was supported in a recent literature review that suggested “face length, face width, and lip length have not been shown to be consistently correlated with fit factor”, suggesting that there is no consensus on facial dimensions and a successful mask fit^7^.

Traditional methods of facial measurements, for example, the bivariate models, have relied on manual landmarking and measurement of specific distances and angles directly on an individual’s face^11^. The validity of using inter-landmark distances, based on the Euclidian distance, has previously been questioned^12^. For example, an increase in Euclidian distance between nasion and the nasal tip could be the result of the nose becoming more pointed, with just the tip moving forward or because of an overall larger nose. More importantly, the complexity of facial shape and facial surface topography cannot be reconstructed based on just four landmarks and two inter-landmark distances. From a design and manufacturing perspective knowing the surface topography and projection of the face is of greater importance when “fitting” a mask to the facial surface rather than specific anthropometric measurements. The routine ease of 3D capture and analysis now make it possible to design masks customised directly on the digital facial surface using CAD/CAM technology^13^.

The aims of this study were to determine which facial characteristics were statistically significantly different between a group of individuals who passed on at least four different FFP3 masks (pass group) and a group of individuals who passed on only one type of FFP3 mask (fail group). The null hypothesis was that there were no statistically significant differences (*p* < 0.05) in facial characteristics between the pass group and the fail group.

## Materials and methods

### Study setting

This study was undertaken at the Birmingham Dental Hospital, Birmingham Community NHS Healthcare with the University of Birmingham. Ethics approval was obtained by the University of Birmingham Science, Technology, Engineering and Mathematics Ethical Review Committee (ERN_21–0610). The study followed the STROBE checklist (https://www.strobe-statement.org/checklists/).

### Sample size calculation

To detect a 3.0 mm difference in a facial dimension between the pass and fail group groups, based on a power of 80%, a statistical significance level of 0.05, with a standard deviation of 3.5 mm (effect size = 0.86), a minimum sample size of 23 individuals in each group needed to be recruited^7^.

### Recruitment

Quantitative mask fit testing using a PortaCount^®^ Respirator Fit Tester (TSI Incorporated, Minnesota, USA) was carried out by trained individuals from the Birmingham Community Healthcare NHS Foundation Trust. The “pass group” comprised of 25 consecutive participants who passed on a minimum of four different FFP3 masks. The “fail group” comprised 25 consecutive participants who passed on only one type of FFP3 mask.

### Imaging technique

All participants were imaged using the passive stereophotogrammetry DI4D SNAP 6200 camera system (Dimensional Imaging Ltd., Glasgow, Scotland). The system was calibrated according to the manufacturer’s instructions. Calibration involved imaging a target with circles with known distances between their centres at 6 different orientations. Using the principle of triangulation and the manufactures calibration software it was possible to determine the camera systems intrinsic and extrinsic properties, and 3D depth of capture. The accuracy of facial capture of the system has been previously reported and has been used extensively over the last twenty years in clinical practice. Before image capture, participants were instructed on how to perform and rehearsed two facial expressions – rest and maximum smile. Rest compromised of teeth in occlusion at maximum intercuspation with lips in repose. Images taken at maximum smile involved the participant in maximum intercuspation with a lips apart full smile. Once ready for imaging, the participant was seated on a chair with the blue screen behind them, facing the camera system. Any glasses or jewellery were removed, and a surgical cap was donned to ensure that no facial hair was obscuring or crossing the participants’ face. The subjects’ 3D images were saved as a Dimensional Imaging binary session (.DI3D) file and converted to a Wavefront Object file (.OBJ) file for later analysis. In total, four groups were created; a pass and fail group consisting of 2 facial expressions – rest and maximum smile.

### Landmarking

**Fig. 1:**
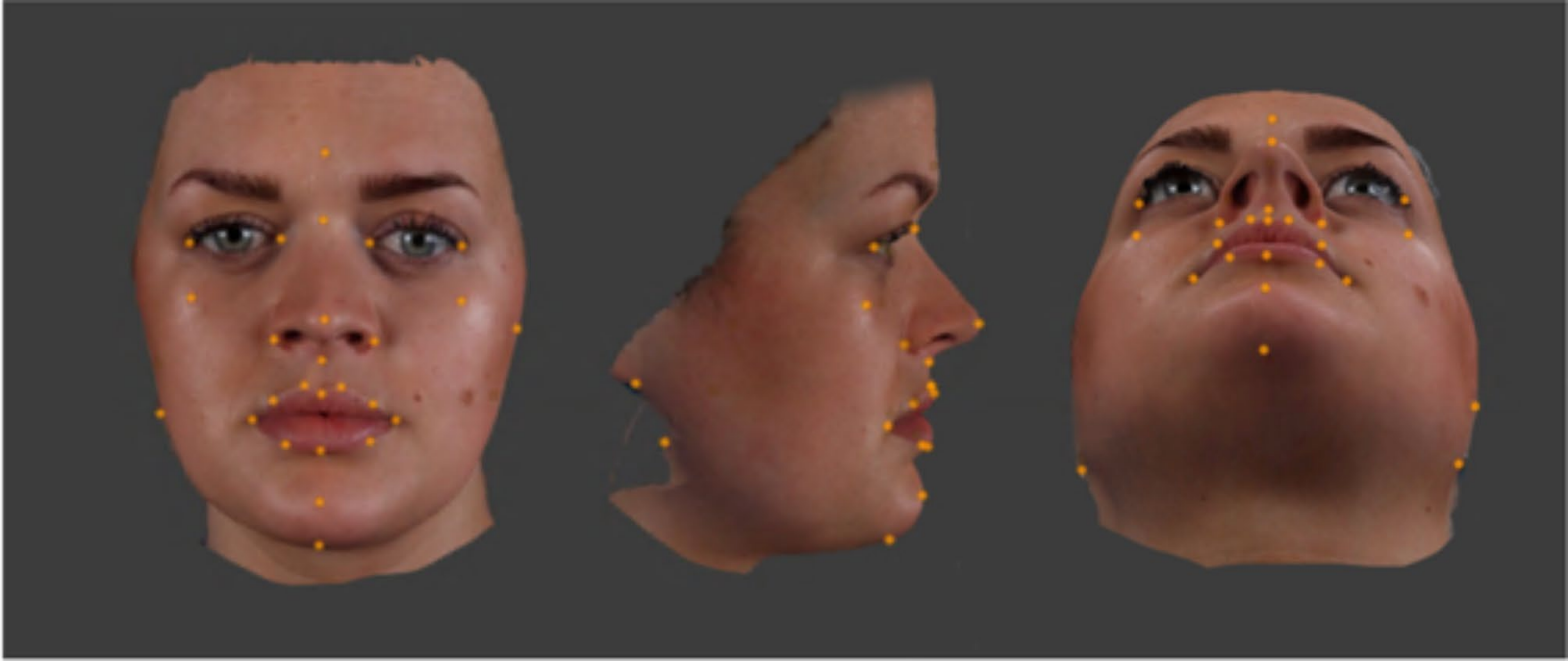
Twenty-eight landmarks placed on the Di3Dview 3D facial image.

For each participant, the .OBJ 3D image at rest was imported into Di3Dview. Twenty-eight landmarks were placed on the 3D facial image (Fig. 1), and the x, y and z coordinates of each landmark were saved as a Dimensional Imaging Landmark (.DILM) file. The landmarks are defined in Table 1. Di3Dview allowed each 3D facial image to be viewed at three different orientations simultaneously – frontal view, profile view and view from below. This allowed accurate landmark placement on the image in all three planes of space. This was repeated for the maximum smile images. Some of the 28 landmarks were used to determine linear and angular measurements, and all were used in creating an “average face”.

**Table 1:** Landmarks placed on the 3D facial image.

Landmark number	Landmark name	Landmark description
1 and 4	Exocanthion	Lateral point of palpebral fissure, right and left
2 and 3	Endocanthion	Medial point of eye fissure, right and left
5 and 7	Ala base	Lateral portion of each alar curvature, right and left
6	Pronasale	Furthest anterior extension of the nose (tip of the nose)
8 and 14	Chelion	The point located at each labial commissure, right and left
9	Mid-point between points right chelion and right christa philtre	Point mid-way between right chelion and right christa philtre
10 and 12	Christa philtre	Point above vermillion border on crest of philtrum, left and right
11	Philtrum of lip	Mid-point of upper vermillion border
13	Mid-point between points left christa philtre and left chelion	Point mid-way between left chelion and left christa philtre
15	Midpoint between left chelion and labrale inferius	Point mid-way between left chelion and labrale inferius
16	Labrale inferius	Mid-point of lower vermillion border
17	Mid-point between right chelion and labrale inferius	Point mid-way between right chelion and labrale inferius
18	Pogonion	Most anterior point on the anterior surface of chin in the sagittal plane
19	Nasion	Mid-point on the soft tissue contour of the base of the nasal root at the level of the frontonasal suture
20 and 21	Preauricular region	Point where skin of the ear merges with skin of face, right and left
22	Subnasale	Point in sagittal view where nasal septum and upper lip merge
23	Glabella	Anterior point on forehead in the mid-sagittal plane
24	Menton	Most inferior mid-point of chin
25 and 26	Zygoma	Point where ala base and exocanthion intersect, right and left
27 and 28	Gonion	Lateral point on the soft tissue contour of each mandibular angle right and left

### Error study

After 2 weeks, the reliability of the methodology was assessed; 20 3D facial images were chosen at random and re-landmarked by the same individual (MKG). The differences in mean absolute distance in the x, y and z directions between the two digitisations were assessed.

### Average face construction

**Fig. 2:**
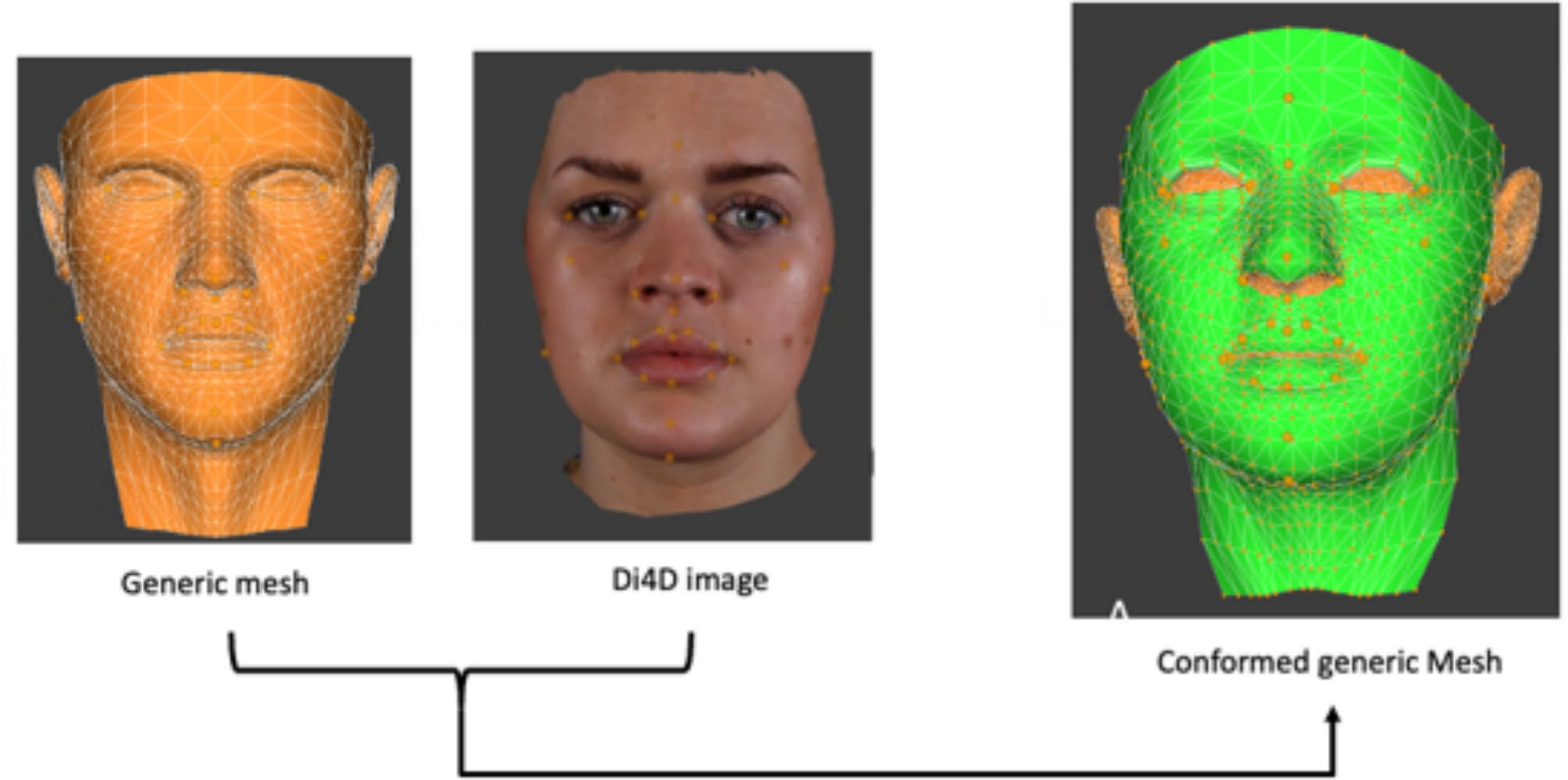
Conformation process.

A perfectly symmetrical facial mesh (generic mesh) made up of 1483 vertices and 2966 triangles / faces was used for the conformation process. For each subject, the captured 3D facial mesh and generic mesh were imported into DiView, the same corresponding twenty eight landmarks were placed on both meshes, Table 1. Using the “shape transfer” function in DiView, the 28 landmarks on the generic mesh were warped to the exact position of the corresponding landmark on the volunteer’s own facial 3D mesh. All of the vertices of the warped generic mesh were then projected along the surface normal onto the surface of the 3D facial mesh, giving the generic mesh the exact shape of the 3D facial mesh (Fig. 2)^14^. For each of the four groups the relevant conformed generic meshes were saved in a separate folder. An average 3D facial mesh surface based on the mean position of each correspondence of all individuals was created using the “Average Face” function in Di3DView. This was saved in .OBJ format.

## Analysis

The data was tested for normality using the Shapiro–Wilk test and was found to be normally distributed for linear, geodesic, and angular measurements. The data was based on continuous values and the variances for the two independent groups were equal. Therefore an independent two-sample *t*-test was used to determine whether there were statistically significant (*p* < 0.05) difference in the mean between the two groups. For volume and surface area enclosed within the mask the data was found to be non-normally distributed. As the data was continuous a Mann-Whitney U test was used to determine whether there was a statistically significant (*p* < 0.05) difference in the median between the two groups.

### Linear and angular measurements

The .DILM file containing the x, y, z coordinates of the landmarks was imported into EXCEL, and the Euclidian distance and angles between the relevant landmarks were calculated. In total, 12 linear measurements and 5 angles were calculated for all the individuals in each of the four groups.

### Geodesic distances

**Fig. 3:**
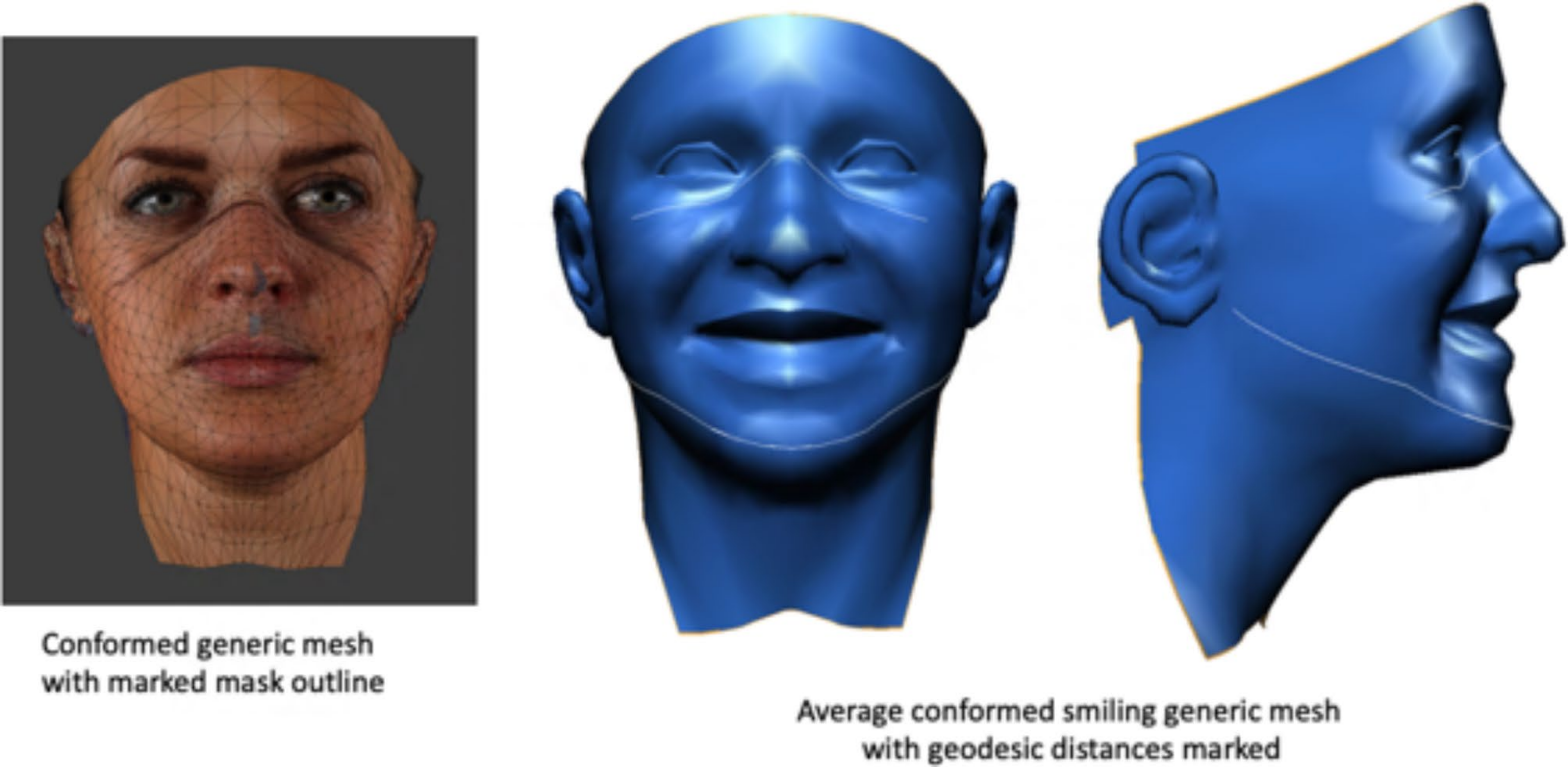
Geodesic distances of the upper mask boundary and mandibular outline.

One individual from each group was chosen at random and fitted with an FFP3 mask. The outline of the FFP3 was drawn onto the individual’s face using an eyeliner pencil. The subject was then imaged using Di4D SNAP as previously described. The 3D image was used to conform the same generic mesh as previously discussed. Using the “material transfer” function in Di3Dview, the original photorealistic colour facial soft tissue was transferred to the conformed generic mesh. This process allowed identification of the vertices, which were on or close to the boundary of the mask (Fig. 3).

For each individual, the conformed generic mesh was imported into Meshlab and converted to .PLY format^15^. Using MATLAB software (MATLAB and Statistics Toolbox Release 2021b, The Math-Works, Inc., Massachusetts, USA), code was written to calculate the geodesic distance of the upper mask boundary and mandibular outline using the vertices previously identified. Again, this was carried out for the four groups.

### Volume and surface area

**Fig. 4:**
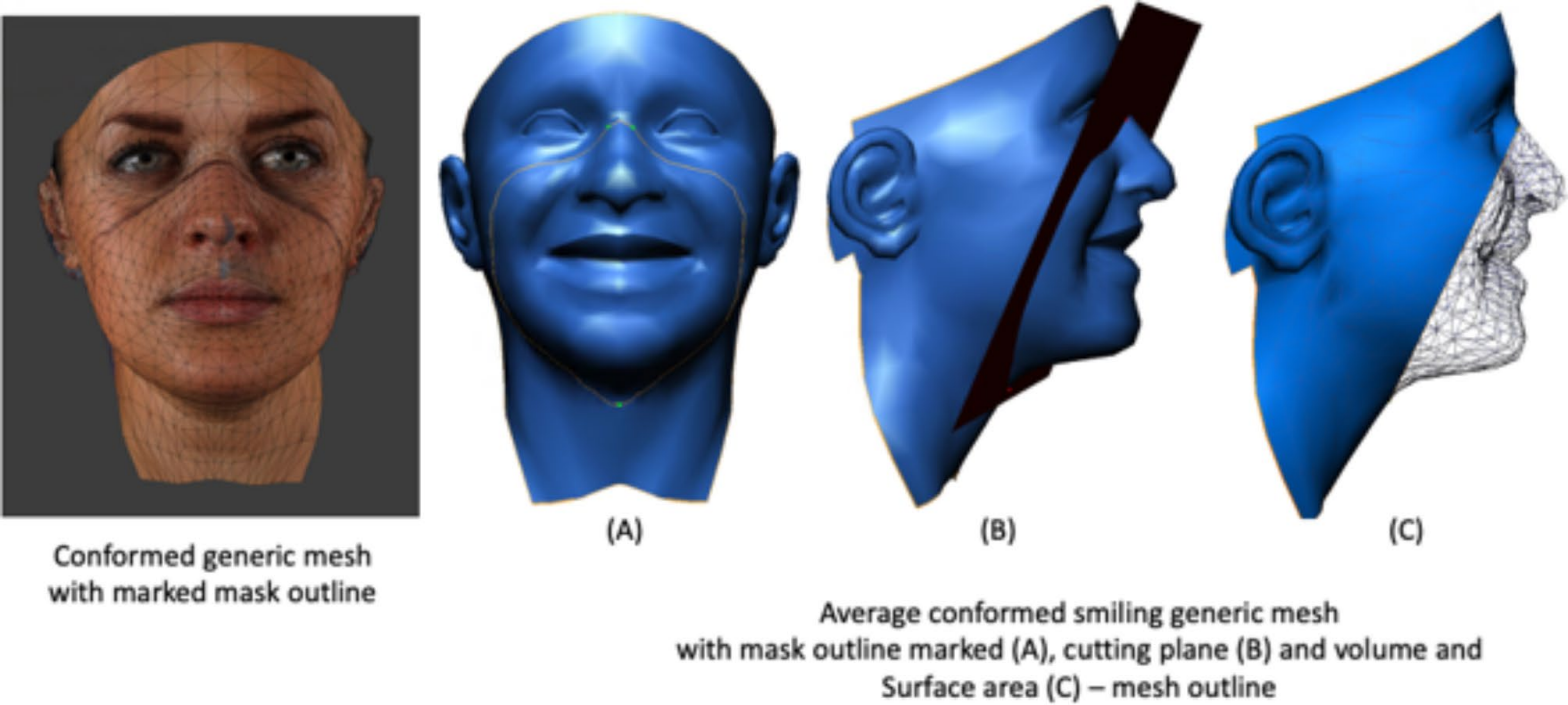
Volume and surface area of the facial surface anterior to the plane, which was enclosed within the mask.

For each individual, the conformed generic mesh was imported into 3dMD Patient software (3dMD Inc., Atlanta, GA, USA). A plane was oriented to pass through the vertices representing the mask outline. The same vertices were used across all the images. The software then calculated the volume and surface area of the facial surface anterior to the plane, which was enclosed within the mask (Fig. 4). This was carried out for all the images in the four groups.

### Average face superimposition

Using Di3Dview, each of the four average faces were orientated so the x axis (x-z plane) was parallel to the inter-pupillary line and the Frankfort plane, the y axis (y-z plane) was set to pass through the mid-facial axis at soft tissue Nasion and was perpendicular to the x-z plane. Finally, the z axis (x-y plane) was set to pass though right and left tragus. Following this the pass group at rest and fail group at rest 3D images were aligned on the forehead/inter-canthal region using only image translation, with no rotation or scaling. Colour maps were produced to show the distances between the two average face surfaces. This was repeated for the average pass group at maximum smile and fail group at maximum smile facial images.

## Results

### Sample demographics

In total, 50 volunteers were recruited for the study, comprising of 10 males and 40 females.

### Error study

After 2 weeks, the reliability of the methodology was assessed; 20 3D facial images were chosen at random and re-landmarked. The differences in mean absolute distance in the x, y and z directions between the two digitisations were assessed. No systematic errors were noted, and all coefficients of reliability were greater than the 90% threshold. The overall mean absolute measurement error was 0.27 ± 0.64 mm in the x-direction, 0.27 ± 0.56 mm in the y-direction, and 0.26 ± 0.60 mm in the z-direction.

### Linear and angular measurements

The mean differences in the 12 linear and 5 angular measurements between the pass and fail groups at rest are shown in Table 2. The only statistically and clinically significant differences were in total face height and mouth width. The mean differences in mouth width and total face height were 2.1 mm (95% CI for the mean difference of 0.2 to 4.0 mm, effect size = 0.64) and 4.5 mm (95% CI for the mean difference of 0.7 to 8.4 mm, effect size = 0.67), respectively.

**Table 2:** Descriptive statistics for actual and differences in clinical measurements between the pass and fail groups at rest.

	Pass group				Fail group				Pass–fail group			
	Mean (mm)	SD (mm)	95% CI (mm)		Mean (mm)	SD (mm)	95% CI (mm)		Mean difference (mm)	95% CI for the mean difference (mm)		*p*-value
			Lower	Upper			Lower	Upper		Lower	Upper	
Linear measurements												
Alar base	34.7	3.1	33.4	35.6	33.7	2.3	32.7	34.6	1.0	− 0.6	2.6	0.200
Bigonian width	118.4	9.7	114.4	122.4	112.0	14.3	106.1	118.0	6.4	− 0.6	13.3	0.073
Bizygoma width	88.1	5.3	86.0	90.3	87.3	3.4	85.9	88.7	0.8	− 1.7	2.3	0.523
Bitragus width	135.2	7.4	132.2	138.2	131.7	10.5	127.4	136.0	3.5	− 1.7	8.6	0.185
Exo(R)–Exo(L)	89.4	5.0	87.4	91.5	88.3	3.3	86.9	89.7	1.1	− 1.3	3.6	0.341
Nasal root width	31.4	3.3	30.0	32.8	31.1	2.6	30.0	32.2	0.3	− 1.4	1.9	0.766
Mouth height	16.5	3.2	15.2	17.8	15.3	3.6	13.8	16.8	1.2	− 0.7	3.2	0.221
Mouth width	52.1	3.0	50.8	53.3	50.0	3.6	48.5	51.5	2.1	0.2	4.0	**0.029** ^*a^
Nose length	51.5	2.9	50.3	52.6	50.7	3.1	49.4	52.0	0.8	− 1.0	2.5	0.379
Nose protrusion	19.2	1.8	18.4	19.9	19.5	1.2	19.0	20.0	− 0.3	− 1.2	0.6	0.528
Upper lip length	14.5	2.5	13.5	15.6	13.6	2.9	12.5	14.8	1.0	− 0.6	2.6	0.200
UAFH	70.0	4.7	68.1	72.0	68.7	3.9	67.1	70.3	1.3	− 1.2	3.8	0.289
LAFH	68.5	5.1	66.4	70.7	65.7	6.6	63.0	68.4	2.8	− 0.5	6.3	0.094
TAFH	136.1	6.2	133.6	138.7	131.6	7.2	128.6	134.5	4.5	0.7	8.4	**0.020** ^*a^
Angular measurements												
Ac(R)-Prn-Ac(L)	66.3	7.4	63.2	69.3	64.4	4.9	62.4	66.4	1.9	− 1.7	5.4	0.304
Ex(R)-N-Ex(L)	129.1	6.8	126.4	132.2	129.9	4.8	128.0	132.1	− 0.8	− 4.2	2.6	0.630
N-Prn-Pog	131.0	4.6	129.1	132.9	128.9	5.1	126.8	131.0	2.1	− 0.7	4.8	0.142
N-S-Pog	164.4	5.5	162.2	166.8	163.5	7.9	160.2	166.7	0.9	− 2.9	4.9	0.625
Sbtr(R)-Sn-Stbr(L)	73.2	4.9	71.2	75.2	74.5	6.0	72.0	77.0	− 1.3	− 4.4	1.8	0.407

*CI* confidence interval, *SD* standard deviation.

*Statistically significant at p value < 0.05.

^a^Paired samples *t*-test.

At the maximum smile, statistically and clinically significant differences (*p* < 0.05) were observed in the upper anterior face height, mouth width and upper lip length. The mean differences in upper anterior face height, mouth width and upper lip length were 5.4 mm (95% CI for the mean difference of 1.1 to 9.6 mm, effect size = 0.26), 3.3 mm (95% CI for the mean difference of 0.9 to 5.7 mm, effect size = 0.78) and 4.0 mm (95% CI for the mean difference of 0.7 to 7.2 mm, effect size = 0.18) respectively, Table 3.

**Table 3:** Descriptive statistics for actual and differences in clinical measurements between the pass and fail groups at maximum smile.

	Pass group				Fail group				Pass–fail group			
	Mean (mm)	SD (mm)	95% CI (mm)		Mean (mm)	SD (mm)	95% CI (mm)		Mean difference (mm)	95% CI for the mean difference (mm)		*p*-value
			Lower	Upper			Lower	Upper		Lower	Upper	
Linear measurements												
Alar base	39.4	3.6	38.0	41.0	37.8	2.9	36.6	39.1	1.6	− 0.3	3.5	0.089
Bigonian width	117.7	10.9	113.2	122.2	113.2	14.6	107.1	119.2	4.5	− 2.8	11.8	0.223
Bizygoma width	86.6	4.6	84.8	88.5	86.1	3.3	84.7	87.5	0.5	− 1.7	2.8	0.653
Bitragus width	135.5	6.7	132.7	138.3	131.9	10.3	127.7	136.2	3.6	− 1.4	8.5	0.157
Exo(R)-Exo(L)	88.3	4.7	86.4	90.2	87.3	3.4	85.9	88.7	1.0	− 1.4	3.4	0.394
Nasal root width	32.7	3.8	32.2	34.3	32.5	3.6	31.0	34.0	0.2	− 1.9	2.3	0.852
Mouth height	24.7	3.7	23.2	26.2	22.5	4.2	20.8	24.3	2.2	− 0.1	4.5	0.059
Mouth width	65.2	4.0	63.6	33.8	61.9	4.5	60.1	63.8	3.3	0.9	5.7	**0.009** ^*a^
Nose length	50.5	3.5	49.1	52.0	50.7	4.0	49.1	52.4	− 0.2	− 2.3	2.0	0.860
Nose protrusion	20.6	2.7	19.5	21.7	20.8	2.2	19.8	21.7	1.1	− 1.4	3.5	0.380
Upper lip length	10.4	2.3	9.4	11.3	10.0	2.3	8.8	11.3	4.0	0.7	7.2	**0.019** ^*a^
UAFH	68.8	4.4	67.0	70.6	67.7	4.1	66.0	69.4	5.4	1.1	9.6	**0.015** ^*a^
LAFH	70.7	5.3	68.5	72.9	66.7	6.1	64.2	69.3	1.6	− 0.3	3.5	0.089
TAFH	138.2	7.3	135.2	141.2	132.8	7.6	129.7	136.0	4.5	− 2.8	11.8	0.223
Angular measurements												
Ac(R)-Prn-Ac(L)	73.7	8.0	70.5	77.0	70.1	6.4	67.5	72.8	3.6	− 0.5	7.8	0.084
Ex(R)-N-Ex(L)	132.1	7.8	128.6	135.2	132.0	5.9	129.7	134.7	0.1	− 3.8	4.1	0.933
N-Prn-Pog	132.2	5.0	130.2	134.3	131.1	5.0	129.1	133.2	1.1	− 1.8	3.9	0.455
N-S-Pog	169.5	5.6	167.2	171.8	168.2	7.4	165.1	171.2	1.3	− 2.4	5.1	0.480
Sbtr(R)-Sn-Stbr(L)	74.7	4.3	72.9	76.5	76.0	6.7	73.2	78.7	− 1.3	− 4.5	2.0	0.435

*CI* confidence interval, *SD* standard deviation.

*Statistically significant at p value < 0.05.

^a^Paired samples *t*-test.

### Geodesic distances

The mandibular geodesic distance was statistically significantly larger by 1.3 cm (95% CI for the difference 0.4 to 2.3 cm) in the pass group at rest than in the fail group (*p* = 0.009), Table 4. At maximum smile, a difference of 1.5 cm (95% CI for the difference 0.3 to 2.7 cm) was also statistically significantly (*p* = 0.017) larger in the pass group. For the nasal bridge including the cheeks, there were no statistically significant differences between the two groups at rest or at maximum smile.

**Table 4:** Difference in volume and surface area of soft tissue in the mask region and nose bridge/cheek and mandibular geodesic distances at rest and maximum smile for the pass and fail groups.

Group	Volume of soft tissue in mask region (cm^3^)	Surface area of soft tissue in mask region (cm^2^)	Nose bridge and cheek geodesic distance (cm)		Mandibular outline geodesic distance (cm)	
	Median	Median	Mean	SD	Mean	SD
Pass rest	451.6	164.1	12.2	0.6	27.3	1.4
Fail rest	385.7	157.7	12.1	0.6	26.0	2.0
Pass smiling	434.3	173.6	12.2	0.6	28.0	2.1
Fail smiling	401.4	167.5	12.1	0.7	26.5	2.1

### Volume and surface area

Following a Mann-Whitney U test, the median difference in volume (50.6 cm^3^) enclosed within the mask at rest was found to be statistically significantly larger (*p* = 0.046) in the pass group than in the fail group, shown in Table 4. The 95% confidence level for the median difference ranged from 7.0 cm^3^ to 96.9 cm^3^. At maximum smile, the median difference of 37.2 cm^3^ was not statistically significant (*p* = 0.119).

The difference in median facial surface area enclosed within the mask (9.9 cm^2^) was found to be statistically significantly (*p* = 0.048) larger in the pass and fail groups at rest, shown in Table 4. The 95% confidence level for the median difference ranged from 4.7 cm^2^ to 18.1 cm^2^. At maximum smile, the median difference of 8.7 cm^2^ was not significantly different (*p* = 0.119). The 95% confidence level for the median difference was found to range from − 2.2 cm^2^ to 19.0 cm^2^.

### Average face superimposition

**Fig. 5:**
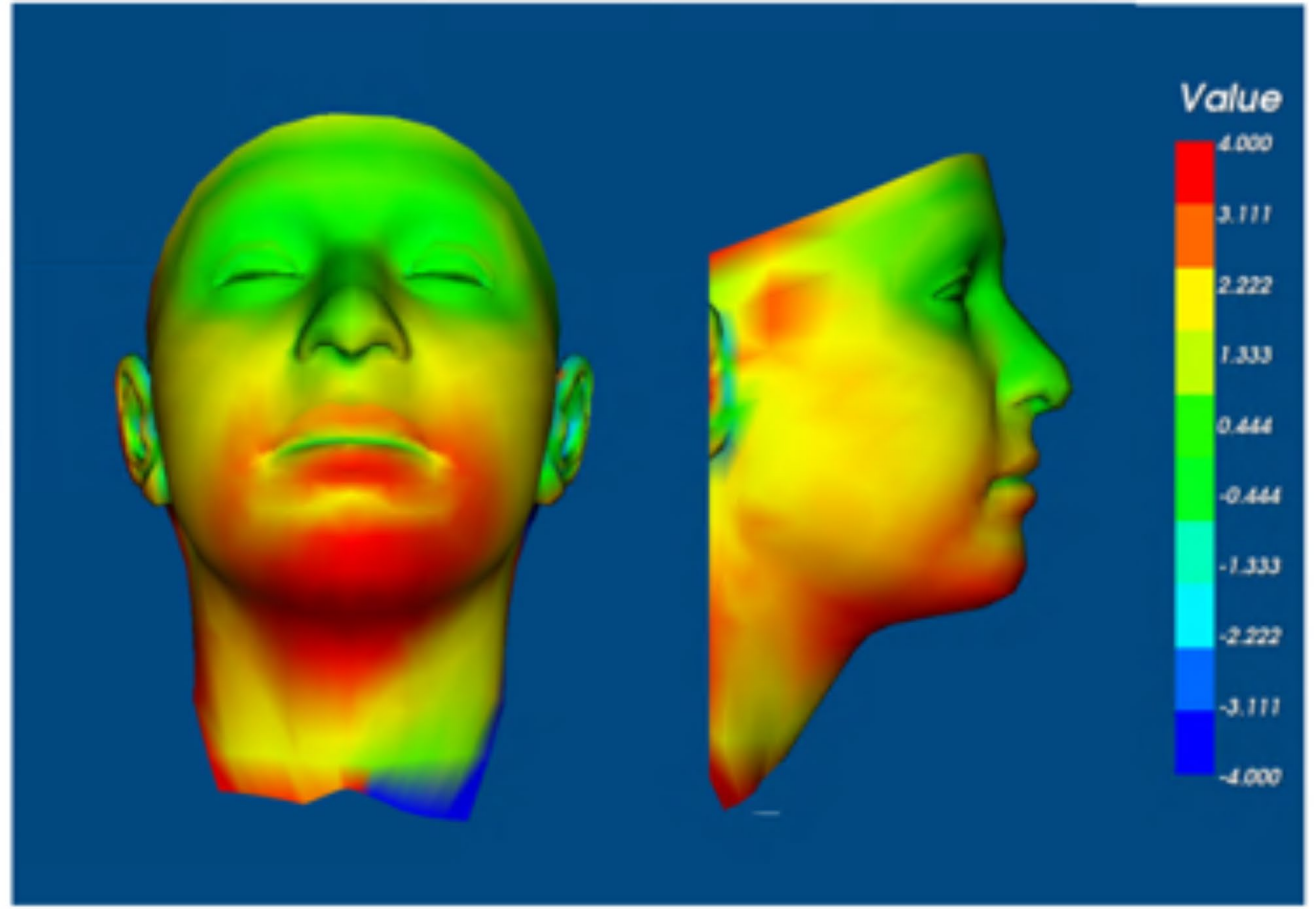
Colour 3D surface distance map between the average pass group and average fail group at rest.

**Fig. 6:**
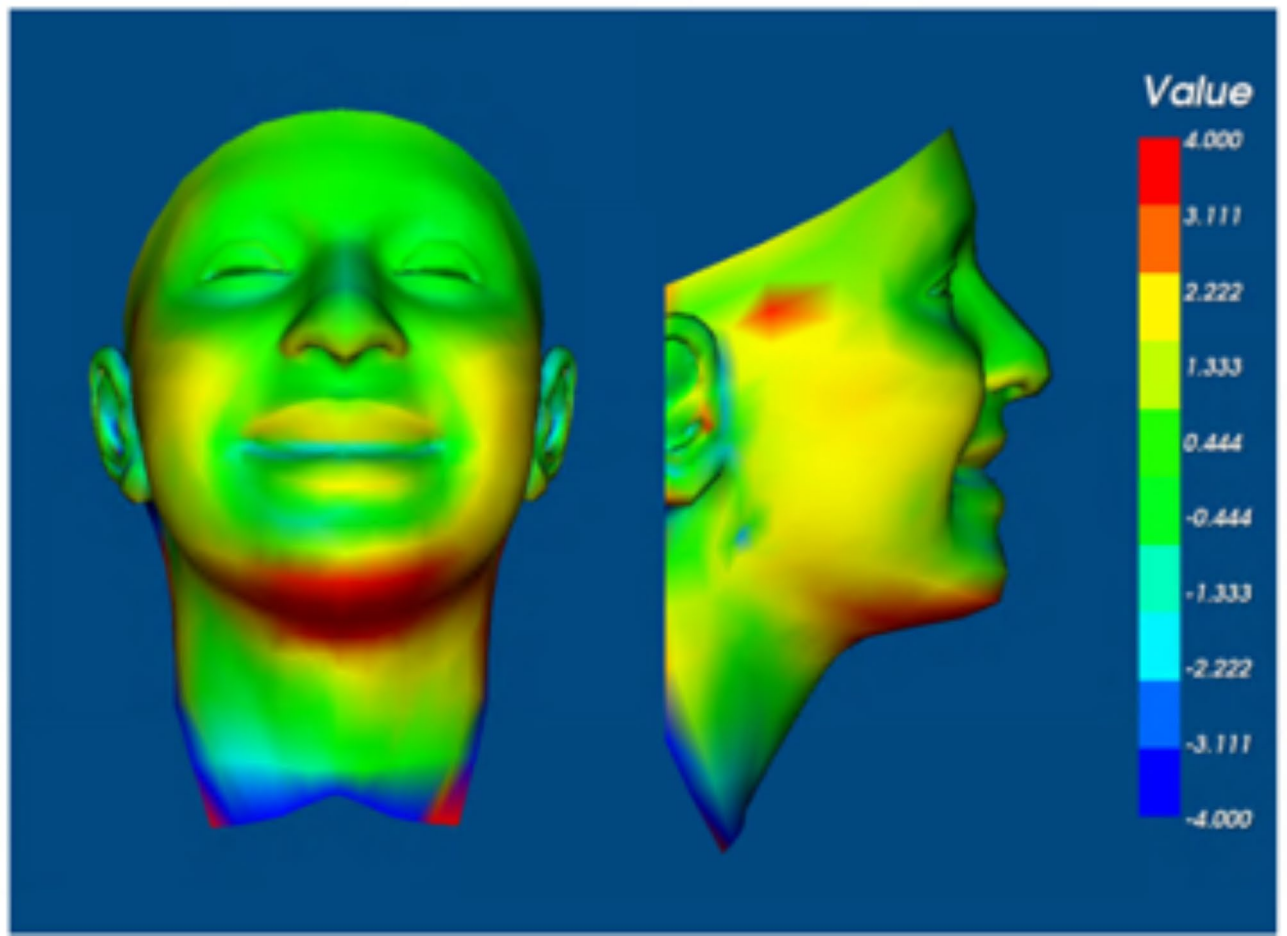
Colour 3D surface distance map between the average pass group and average fail group at maximum smile.

The distances between the average pass group at rest and fail group at rest 3D surfaces were larger than 4.0 mm around the chin as well as around the mouth (Fig. 5). This represents the regions adjacent to the mask periphery. For maximum smile, the distances between the average pass group and fail group 3D facial images were larger than 4.0 mm around the lower border of the mandible and this would coincide with the lower border of the mask (Fig. 6).

## Discussion

This study investigated the differences in facial characteristics between two distinct groups of individuals, a group passing on at least four masks (pass group) and a group passing on only one mask (fail group). All individuals were fitted with the same selection of masks by the same trained fit testers; however, some individuals failed, and some passed. This would suggest differences in the facial dimensions between the two groups. Previous studies have investigated fit factor scores and facial dimensions and have tried to find correlations and predictive variables^5,8,10,16,17^. However, small changes in fit factor scores and subtle facial differences in facial dimensions may not be readily correlated. Other studies have compared the facial anthropometric facial dimensions of different ethnic groups or professions to historical respirator fit test panels^4,9,18–20^. These types of studies are investigating the anthropometric differences between the two cohorts and assuming that any differences in facial dimension would result in the mask not fitting. In most studies, a fit test was not performed to confirm this assumption. Ideally, in the present study, the fail group should have been those individuals who were unable to be fitted on any mask. This would have resulted in a small sample, and the decision was made to choose a group of individuals with minimum pass rates, i.e., one mask, as opposed to total mask failure. However it is acknowledged that not using a total failure to fit group does introduce some bias into the study and is a limitation of the study.

Individuals from ethnic minorities or BAME (Black, Asian, Minority Ethnics) have been shown to have higher risks of failing their fit tests^21^. A recent systematic review and meta-analysis reported that there is limited evidence to conclude that there is an association between ethnicity and successful mask fit^22^. In addition, there is no conclusive evidence supporting gender as a factor that influences the success of fit test passing^22,23^. Although significant differences in facial anthropometrics between males and females were reported, the pass rate for respiratory masks did not differ^23^. However it should be noted that even though ethnicity and gender were not stratified due to inconclusive evidence, facial forms are known to differ. For example based on 3D facial averages, differences between an adult male and female Chinese population and a Houstonian white population have been reported^24^. Houston males showed more prominence in the pogonion, nasal tip, and supra-orbital areas. The Chinese males exhibited wider malar regions and a flatter peri-oral region. Whilst Houston females showed more prominence in the pogonion, nasal tip, and supra-orbital areas, The facial regions which were found to differ were at the either at or in close proximity to the mask boundary and therefore may have an effect on the success of mask fit. This would suggest that mask dimensions should be ethnicity and gender specific. The fact that subjects were not stratified with respect to their ethnicity or gender in the present study may be a limitation of the study. Based on the cohort of staff and student in the establishment it would not be possible to image a large enough sample of each ethnic group.

In the present study, the only linear facial measurements that were statistically significantly larger in the pass group were total face height (TFH) and mouth width. This agreed with previous studies reporting TFH as an important determining factor for a successful mask fit test in a range of populations, including Chinese, Taiwan and South African populations^6,25,26^. The larger mouth width in the pass group has been reported as a deterministic factor of the success of passing the fit test^9^. Interestingly, as a measurement, mouth width should not directly affect mask fit, as it is located well within the mask periphery and is not related to the peripheral facial seal. Mouth width could be an indirect measure of facial width; however the correlation coefficient between mouth width and face width has been shown to be weakly associated^27^. A possible explanation is that those individuals who have a greater mouth width and longer face are likely to be ‘filling’ more of the periphery of the respiratory masks, therefore potentially resulting in a better seal i.e. there is more soft tissue bulk. The present study showed no differences in bi-gonian, bi-zygomatic and bi-tragus width; this is in contrast with the literature^6^. This could be due to the natural large variations in face width seen in the population. For example bigonal width ranged from 90 mm to 160 mm in the male National Institute for Occupational Safety and Health (NIOSH) respirator sample.

An alternative method of assessment, rather than simple straight-line distances and angles, has been the use of 3D facial images and principal component analysis (PCA). Previous studies have used PCA to assess facial dimensions for mask sizing and reported that the first PC describes the overall size of the face and the second PC the face shape, with PC1 and PC2 explaining 36% of the total variation in the facial data^18,19^. The disadvantage of PCA is that some PCs capture differences in only particular parts of the face. Moreover, although the PCs are statistically independent, any particular part of the face is affected by several PCs, making it difficult to identify the contribution of each PC to a clinically relevant regional feature^29^. The present study also used 3D facial surface images but instead of PCA produced a series of “average faces” for analysis. This novel method of assessment creates an average face using a generic mesh and the process of conformation^28^. For the present study, 4 average faces were produced – the average pass face at rest, the average pass face at smiling, the average fail face at rest and the average fail face at smiling. The average face provides a surface that can be used for analysis as well as allowing conventional linear and angular measurements to be taken^11,29^. This methodology also allowed direct comparisons of the 4 different average faces for the volume, surface area and regional differences of the soft tissue enclosed within the mask, as well as cheek and nasal bridge geodesic distances and mandibular outline geodesic distance. The use of linear and angular measurements from average faces has shown to be a valid approach for facial analysis^30^.

**Fig. 7:**
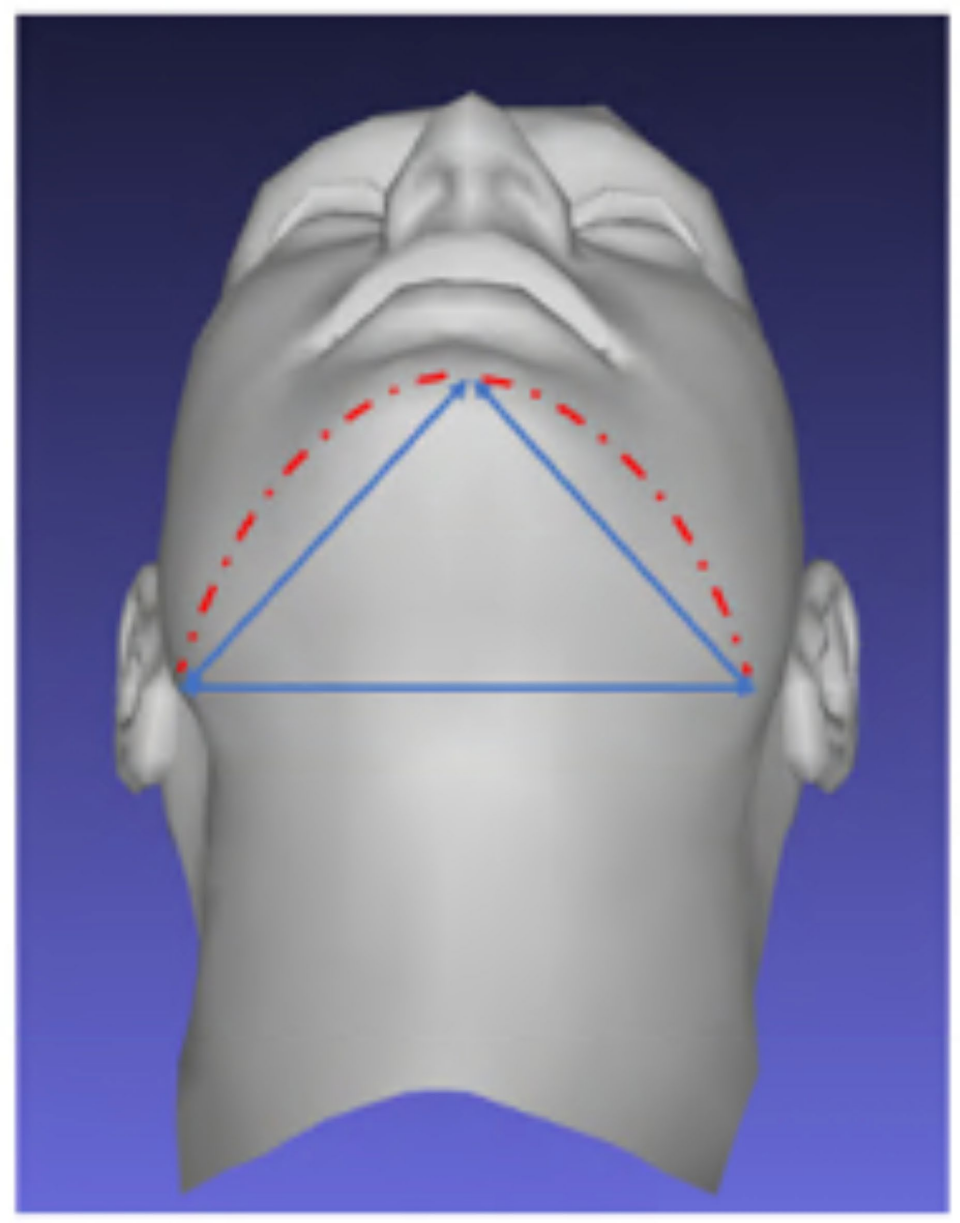
Difference in straight-line distance formed by joining left and right gonion to pogonion and the geodesic distance representing the soft tissue shape to which the mask needs seal against.

The median volume of the soft tissue enclosed within the mask in the pass group at rest was 451.6 cm^3^, while in the fail group, it was 385.7 cm^3^, a median difference of 56.3 cm^3^. The median surface area difference between the pass (164.1 cm^2^) and the fail group (157.5 cm^2^) at rest was 7.4 cm^2^. The greater median volume and surface area in the pass group at rest could be suggestive of more fullness of the soft tissue within the mask region. In the fail group, the fullness of the face may not be enough to fill the mask, hence the lack of a peripheral seal. This conjecture was supported by the findings that the mean mandibular outline geodesic distance was 1.3 cm shorter in the fail group (26.0 cm) than in the pass group (27.3 cm). The use of geodesic or surface distances is more clinically valid when investigating mask fit. For instance, the straight-line distances formed by joining left and right gonion to pogonion does not represent the soft tissue shape to which the mask needs seal against (Fig. 7). The use of geodesic distances at least goes some way to address this shortcoming. These findings suggest that there is not enough facial volume to fit the mask, and that mask periphery needs to be adapted to contour the facial topography.

From rest to maximum smile in the pass group, there was a decrease in median volume, from 451.6 cm^3^ at rest to 434.3 cm^3^ on smiling, but an increase in median surface area from 164.1 cm^2^ at rest to 173.6 cm^2^ on smiling. This might be expected as the soft tissue labial region expands and thins, with an increase in surface area as the cheek region expands laterally and the lower border of the mandibular soft tissue moved superiorly. In the fail group, there was an increase in median volume from 385.7 cm^3^ at rest to 401.4 cm^3^ on smiling and an increase in median surface area from 157.7 cm^2^ at rest to 167.5 cm^2^ on smiling. During smiling, the soft tissue volume of the fail group behaved differently from that of the individuals in the pass group. Interestingly, the median volume of the fail group did not reach the minimum volume of the pass group when at rest or smiling. This may suggest that the fail group does not have the volume of soft tissue bulk to make contact with the mask periphery. This highlights the importance of not just assessing static images but also dynamic facial soft tissue changes. Future research directions should include a more thorough investigation of how facial expressions and facial movements affect mask fit. This is important as currently FFP3 mask development is based on measures from static facial measurements whilst the mask fitting validation process is undertaken whilst undertaking facial expressions and head movement.

The geodesic distances, as well as the facial volume in the soft tissue region of the mask boundary, would indicate that there is more soft tissue bulk, fullness or anterior projection of the face in this region in the pass group than in the fail group. This correlates with the increase in size of the face reported by PCA, as it should translate to an increase in facial volume. When masks are worn and if their fit is not ideal, the wearer is forced to tighten the mask and draw it closer into the face as a means of securing a peripheral seal. A combination of the initial passive mask fit to the facial surface, the amount of soft tissue thickness, the degree of soft tissue compression and the morphology of the bony structures under the soft tissue will all influence how well a peripheral seal can be obtained by drawing the mask tighter into the facial soft tissue. The assumption is that the better the passive fit of the mask is, the less the reliance on the other factors. It is therefore not surprising that in a recent survey of hospital staff, facial pressure injuries from FFP3 masks were reported by nearly 80% of respondents^31^. The amount of facial adipose tissue may affect not only the degree of soft tissue compressibility but also the facial shape. However, body fat proportion has been shown to explain approximately only 9% of the facial shape variation^32^. Given the relatively weak relationship between BMI and facial fat, the BMI of individuals as a variable was not assessed in the present study. True facial soft tissue thickness could have been measured by CBCT scanning, but this would be unethical in this type of study but may be a limitation of the study.

## Conclusions

The null hypothesis was rejected for total facial height, mouth width, mandibular geodesic distance, volume and surface area of the soft tissue enclosed within the mask. These measurements were statistically significantly (*p* < 0.05) larger in the pass group than in the fail group. For the first time, it was reported that the volume and surface area of the facial soft tissue enclosed within the mask are greater, as well as mandibular geodesic distance, in individuals who are likely to pass an FFP3 mask fit test. This would suggest that some masks may be too large for some of the faces, and manufacturers need to reduce the size of their masks or produce masks that accommodate a wider range of facial dimensions, accepting the fact that one size does not fit all. In addition, the mask periphery needs to closely resemble the passive facial contour prior to tightening the mask.

## Data Availability

The four average facial 3D files analysed during the current study available from the corresponding author on reasonable request.
